# Comparison of Cytotoxicity of Three Dentin Bonding Systems with Two Thicknesses of Dentin Barrier on L929 Cell Line

**Published:** 2006-10-01

**Authors:** Farshid MirMotalebi, Shahrzad Nazari

**Affiliations:** 1 *Department of Prosthodontic, Dental School, Hamedan University of Medical Sciences, Hamedan, Iran*; 2 *Department of Endodontic, Dental School, Hamedan University of Medical Sciences, Hamedan, Iran*

**Keywords:** Cytotoxicity, Dentin Barrier, Dentin Bonding Agent

## Abstract

**INTRODUCTION:** Along with introduction of dentin bonding agents (DBA), their clinical use as lining materials is increasing rapidly. Since remaining dentinal thickness (RDT) has always been a concern for cytopathic effect of restorative materials, its effect on reduction of cytotoxicity of these materials especially DBAs is critical. The purpose of this study was to evaluate and compare the cytotoxicity of three dentin bonding systems, belonged to the 4^th^, 5^th^ and 6^th^ generation of DBAs on L929 cell line.

**MATERIALS AND METHODS:** Thirty human premolar teeth were included. Class I cavity preparations were prepared on occlusal surfaces. After crown separation, a flat dentinal surface was provided and RDT (remaining dentinal thickness) was adjusted at 0.5 and 1.5 mm. Then, cavities were treated in three groups with experimental DBAs: Group 1: Scotchbond multipurpose, Group 2: Excite, Group 3: AdheSE. Blue inlay wax sealed the cavities. Crowns were immersed in culture medium for 24 hours and the cytotoxicity of the resultant toxic medium was measured quantitatively with MTT assay in 4 serial dilutions. Data were analyzed with ANOVA and Tukey’s test at 95% significance level.

**RESULTS:** MTT assay determined that only in neat dilution of 0.5 mm RDT, cell changes were significantly different from control. Besides, no significant differences were found between the three experimental DBAs regarding cytotoxic effect on L929 cell line.

**CONCLUSION:** Considering the limitations of an *in vitro* study, if the RDT is less than 0.5 mm *in vivo*, regardless of the type of DBA, destructive cellular changes in pulp tissue can be expected.

## INTRODUCTION

Adhesion was introduced into dental profession following enamel etching by Bounocore in 1955 (1). Dentin, in addition to enamel, is able to provide a suitable substrate for micro- mechanical bonding of restorative materials. Though, this adhesion is much more difficultly achieved due to heterogeneous and complicated nature of dentin.Besides, smear layer formation due to tooth preparation will reduce the penetrability of dentin, which will adversely affect on resin- dentin bond strength.

Improvement in dentin bond strength was achieved through dentin etching. So that, following removal of hydroxyapatite crystals, the collapsed collagen network will re-expand with application of priming agent. Adhesive resin is then applied and cured to from a resin reinforced dentinal layer which was referred as hybrid layer (2).

Literature in the field of cytotoxicity testing of dental materials shows that in older studies, pathologic changes in cell cultures were assessed by counting the number of attached cells or mitosis rate .But, in newer studies, specific cellular parameters are being used and cell density is determined, or as an alternative method, cells are classified as normal, altered or dead (3).

Ratanasathien et al. showed that three specific interactions exist between two monomers based on monomer concentration and duration of interaction: Synergistic, Additive and Antagonistic. So that, after 24 hours, antagonistic effect is more evident which reduces cytotoxicity, but after 72 hours, cytotoxicity is increased because synergistic effect is predominant (4).

Cox and coworkers compared the pulpal response to a number of DBAs and Ca (OH)_2_. They found that with no pulpal exposure, no significant histological change is observed in teeth treated with these liners during short term and long term follow ups. Even with pulpal exposure, no clear histopathological change in DBA treated teeth is seen provided that pulpal hemorrhage is completely controlled (5). The major pulpal response to toxic concentrations of DBA is cell contraction and rounding, coupled with detachment of filliodopia which was observed in two studies (6,7).

In 1999, a study was performed on the cytotoxicity of Single bond, Syntac spirit and Prime & Bond 2.1 applied on immortal MDPC – 23 cell lines. Then cell morphology was evaluated under SEM. The authors revealed that all the experimental DBAs were cytotoxic and acidic and non-acidic components of these DBAs, are responsible for toxicity (8).

In 2001 the effect of Scotchbond multipurpose plus and clearfil LB bond on thyrosine phosphorylation of L929 cell line was evaluated. In this study, the affected cell zone was assessed with an image analyzer and monomer concentration was determined with chromatography after 24 hours incubation time. The authors found HEMA the major released monomer from uncured material followed by a trace amount of TEGDMA. Increasing light curing time, reduced the cytotoxicity, so that, the cured material showed cytotoxicity insufficient for suppression of cell viability (9).

In 2003, a cytotoxic study on four single component dentin bonding systems (One up bond F, Prime & Bond 2.1, Single bond and Syntac) on L929 cell line was conducted. The authors used 0.5 mm thickness dentin discs in a dentin barrier study. Cell viability of all experimental DBAs was reduced to 60% except one up bond F, which only revealed 7% metabolic suppression. Totally, they found total etch bonding systems more toxic than their self etch counterparts (10). In 2005, the selective influence of dentin thickness upon cytotoxicity of Syntac classic, Prompt L- Pop, Vitrebond after 24 hours of exposure time was investigated. MTT assay determined that Syntac Classic decreased Cell activity significantly, independently of dentin thickness. For Prompt L-Pop and Vitrebond a Significant influence of dentin thickness was found on the cell reaction, the authors stated that dentin acts as a barrier, decreasing the elicited cytotoxicity with increasing thickness and the effect is material related (11).

The purposes of this article are 1- comparison of cytotoxicity of experimental DBAs with two dentinal thicknesses, 2- Evaluating the effect of four serial dilutions of these DBAs on their cytotoxicity on L929 cell line.

## MATERIALS AND METHODS

Thirty human premolar teeth, scheduled to be extracted for orthodontic reasons in young individuals were included. Teeth had no sign of carious lesion, attrition or crack lines. After removal of calculus and organic debris, samples were kept in normal saline until being treated.

According to the method described by Camps et al in 1997, class I cavity preparations (4×1.5×1.5mm) were cut in occlusal surfaces of the teeth with high-speed hand piece and diamond fissure bur under air-water spray (12). The crowns were cut at CEJ with a diamond disc and their bottom surfaces were ground so that a flat dentinal surface without any sign of pulp horns could be seen. Then teeth were randomly divided into two 15 sample main groups (A,B).so that, the thickness of the pulpal floor was measured with a gauge and this thickness was adjusted at 0.5mm for group A and 1.5mm for group B. The bottom surfaces of the crowns were conditioned with polyacrylic acid for 10 seconds for smear layer removal.

**Table1:** Chemical composition of three experimental dentin bonding systems

**DBA**		**COMPONET**	**Company**
**Scotchbond multipurpose**	PRIMER	HEMA polycarboxylic acid, water	3M USA
	ADHESIVE	BISGMA, HEMA	
**Excite**	HEMA, dimethacrylatees, phosphoric acid, acrylates		Vivadent Liechtenstein
	Highiy dispersed silicon dioxide		
	Intiators & Stabilizers in alcohol solution		
**Adhe SE**	PRIMER	Dimethacrylates, phosphoric Stabilizersin alcohol solution & acid,acrylates, Intiators	Vivadent Liechtenstein
	ADHESIVE	HEMA, dimethacrylates,silicondioxide, Initiators & stabilizers	

Samples in each group were further subdivided into three subgroups (1, 2 and 3) and treated as follow: Group 1: 37% buffered phosphoric acid was applied to the walls and floor of the cavity for 15 seconds; cavity was rinsed under tap water for 5 seconds and gently air dried, until a frosty enamel appearance was observed. A wet cotton pellet was applied to dentinal surfaces to re-expand the collagen network. Then, according to the manufacture, the primer component of Scotchbond multipurpose DBA (3M, USA) was applied to the cavity surfaces for 20 seconds until a glistering view was observed. The adhesive resin coated the walls and floor and was cured for 20 seconds with a halogen curing device (Astralis 3, Vivadent, Liechtenstein). Group 2: Surface conditioning, rinsing, drying and rewetting the dentinal surfaces were carried out as group 1. Then, according to the manufacture, a single coat of Excite DBA (Vivadent, Liechtenstein), was applied to the cavity walls. A gentle stream of air was applied for 3 seconds to the cavity surfaces for the primer component to be evaporated, and a glistering surface to be observed. The adhesive was cured for 20 seconds. Group 3: According to the manufacture, the primer component of the AdheSE DBA (Vivadent, Liechtenstein) was applied for 15 seconds to the cavity surfaces, rubbed with a brush for additional 15 seconds. A strong stream of air spread the primer completely, immediately followed by the application of the adhesive component. The adhesive was cured for 10 seconds.

A hard blue inlay wax (Kerr, USA) with 40-50º centigrade melting range, was used to seal the cavities. Now samples are ready to be transferred to cell culture laboratory. The chemical composition of the three experimental DBAs is listed in Table 1.

Under laminar hood which can be considered sterile, samples were immersed in 70% ethanol for 10 minutes, exposed to ambient atmosphere for ethanol to be evaporated completely. Then, crowns were placed in centrifugal tubes containing 4 cc of culture medium (DMEM +10% FBS). Tubes were incubated under 37ºc, 100% relative humidity and 5% Co2 for 24 hours. During this time, the uncured monomers were released through dentinal tubules into the culture medium. Thereafter, 1.8 cc of this toxic extract is serially diluted in four dilutions: Neat, ½ v/v, 1/10 v/v, 1/100 v/v.

5000 cells plus 200 µL of culture medium is added in each well of a 96 well cell culture plate. After incubation for 24 hours, culture medium is replaced by the toxic medium with aforementioned 4 serial dilutions, except for the control well. Following 24 hours of exposure time, toxic and culture medium are replaced by MTT dye, which is incubated for 4 hours. 200µL of DMSO and 25µL of buffered glycin are further added to each well. Plates are then shaked at 1500 rpm for 5 minutes, in order to formazan crystals be completely solved. The optic density (OD) of the resultant suspension is measured with an Elisa Reader device (Stateax, USA) at 540 nm wavelength. Finally, the OD data were statistically analyzed with ANOVA and Tukey’s tests with p-value was adjusted at 0.05.

## RESULTS

**Figure 1:**
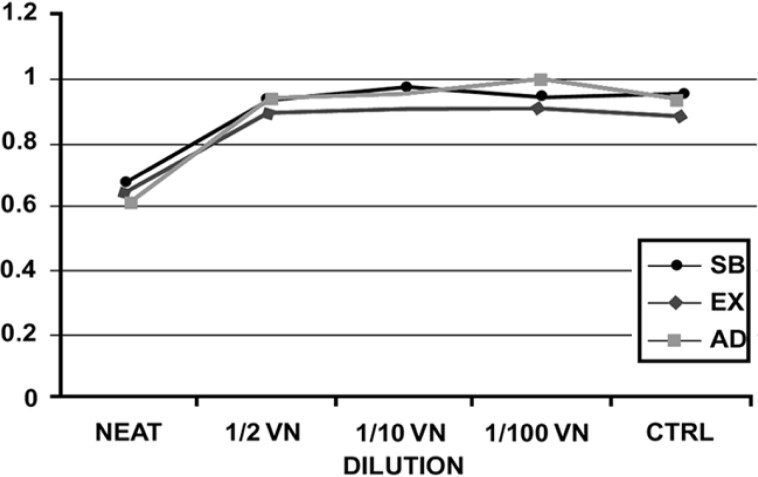
The mean OD values of group A (0.5 mm RDT) (Sb: scotchbond, Ex: excite, Ad: adheSE)

**Figure 2:**
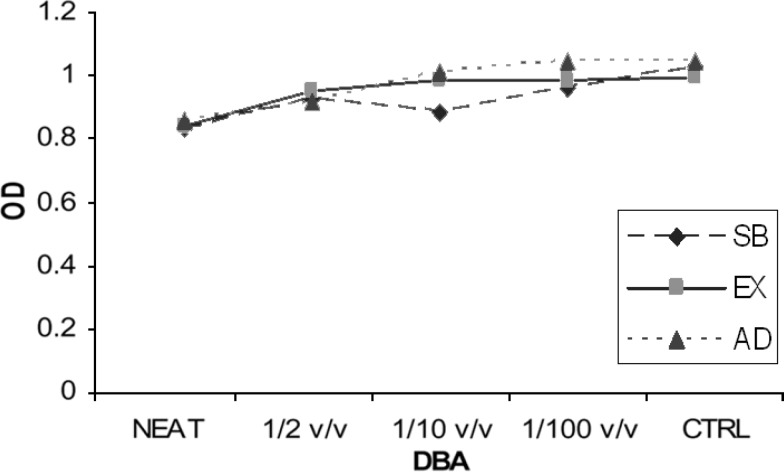
The mean OD values of group B (1.5 mm RDT) (Sb: scotchbond, Ex: excite, Ad: adheSE

**Figure 3:**
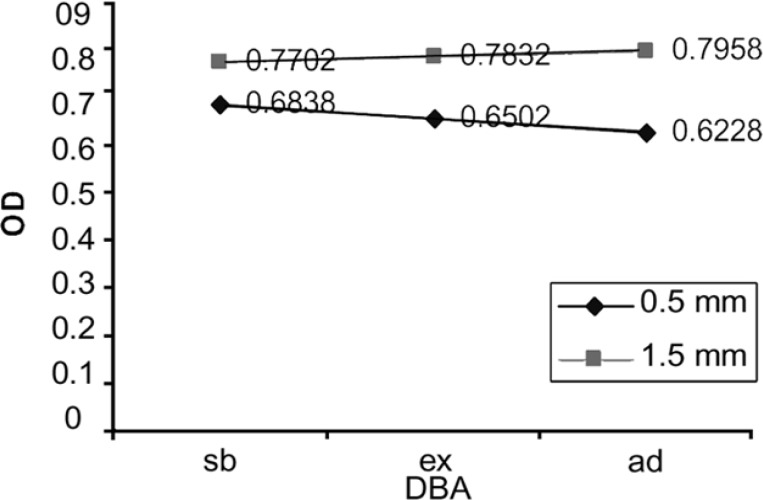
The mean OD values of six subgroups based on dentin thickness. (Sb: scotchbond, Ex: excite, Ad: adheSE)

**Figure 4:**
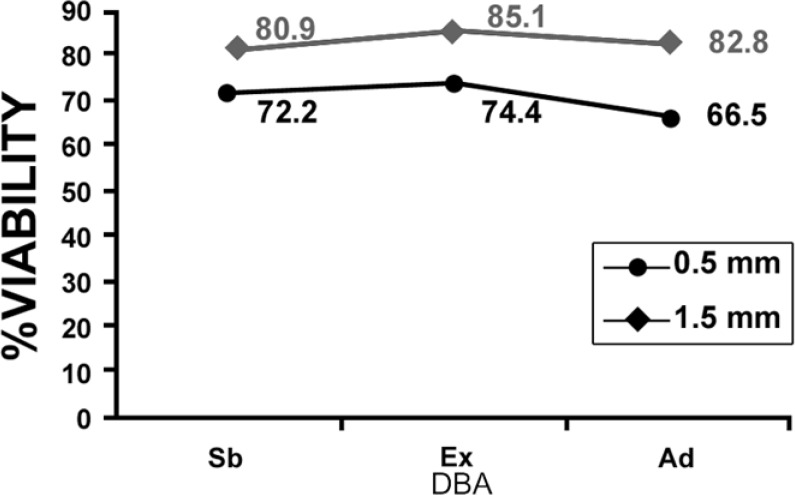
The percent viability of L929 cells in experimental subgroups (Sb: scotchbond, Ex: excite, Ad: adheSE)

The mean values of the OD for each of two experimental groups are presented in Figure 1, Figure 2, and Figure 3 shows the OD mean values of six subgroups based on two dentin thicknesses and the percentage of cell viability can be observed in Figure 4. Data analysis revealed that: 1) in group A, the mean value of OD in neat dilution is significantly higher than, 1/2, 1/10, 1/100 v/v dilutions and control group. 2) For each subgroup, no significant difference was found among, 1/2, 1/10 and 1/100 v/v dilutions or between these dilutions and control group. (p=0.513). 3) No significant difference was found between the OD mean values of three experimental neat dilutions (P=0.436). 4) No significant difference was found between the OD mean values of the three experimental subgroups in 1/2 v/v dilution (P=0.540), 1/10 v/v dilution (p=0.543) and 1/100 v/v dilution (P=0.314). 5) Cell metabolism suppression values in neat dilution were 27.8%, 25.6% and 33.5% for subgroups 1, 2 and 3 respectively.

## DISCUSSION

Cytotoxicity tests of dental materials have undergone significant evolution through the years. Recently these tests, utilize a dentin barrier model for better *in vivo* simulation. Two main methods are commonly used to provide a dentin barrier *in vitro*: a) tooth crown is sectioned horizontally to produce dentin discs with the desired thickness, b) the method which was first described by Camps et al. in 1997, which we applied in our study (12).

We applied minor deviations from that study. First, we used intact human extracted premolar teeth instead of molars. Since, these teeth are more routinely extracted in young individuals for orthodontic purposes. Second, we prepared smaller cavities according to the smaller size of premolars.

A pilot study was included in the study to compare the efficacy of 70% ethanol versus autoclave in disinfecting the samples. Evidently, cell culture tests should be performed in an isolated microorganism free condition. Any contamination present in experimental materials, plates or ambient atmosphere can lead to failure. Although, autoclave is used more routinely to sterilize the samples in dentin barrier studies, but, some particular researchers have used 70% ethanol for sample disinfection (12). So, we decided to compare the suitability of these two methods for cytotoxicity study. Based on the results, two methods are equally efficient to provide contamination free situations. Therefore, since samples can be disinfected in ethanol directly under laminar hood and then immediately immersed in culture medium in an absolute sterile condition, the 70% ethanol disinfection method was selected.

Time plays a major role in cytotoxicity of dental materials. In this study, 24 hours for sample exposure to the culture medium was allowed. This exposure time is in accordance with at least six studies which measured the cytotoxicity of dentin bonding systems on L929 Cell line. (9-14)

L929 Cell line was selected to measure the cytotoxicity of experimental materials. Literature reveals that this cell line is more frequently used for *in* *vitro* cytotoxicity tests than other cell lines (9-14).

It is clear that continuous cell lines are more suitable for short term studies. Since, these cells have undergone numerous cycles of passage; they can better express their characteristics persistently. This in turn will increase the validity of the results. On the other hand, in longer term studies, primary cell lines can better simulate *in vivo.* This is due to their ability to exhibit physiologic conditions like aging and unstable mitosis (15).

An important consideration about this study is the absence of simulating pulpal pressure. The explanation to this is that in other studies which apply pulpal pressure, an *in vitro* pulp chamber device is utilized. This device was not affordable for this study.

Based on the results of this study, only in 0.5 mm dentin barrier group, the three experimental DBAs showed significantly more cytotoxic effect in neat dilution but, in 1/2, 1/10 and 1/100 v/v dilutions, these DBAs did not show significantly more cytotoxicity than culture medium. Although some cytotoxic changes could be seen in cell morphology but this observation could not be validated through statistical analysis. So, we can declare that 0.5 mm remaining dentinal thickness permits passage of uncured monomers toward the pulp. Another important finding of this study was that diluting the toxic medium even to 1/2 v/v, can significantly reduce the cytotoxic effect of the studied DBAs on L929 cell line. This is in agreement with at least two studies (13,16).

Besides, it was revealed that there was no significant difference between Scotchbond multipurpose, Excite and AdheSE bonding systems regarding cytotoxicity. This can be important, since each of these DBAs belong to a separate generation with rather different composition and mechanism of action.

## CONCLUSION

1- Scotchbond multipurpose, Excite and AdheSE bonding systems showed similar cytotoxicity effects on mouse fibroblasts.

2- Only neat dilution of these DBAs, could express cytotoxic effects via a 0.5 mm thickness dentin barrier.

3- Clinically, degenerative cellular changes in pulp tissue can be expected provided that RDT is equal or less than 0.5 mm.
